# Lives of Eminent American Physicians and Surgeons of the Nineteenth Century

**Published:** 1861-05

**Authors:** 


ART. II.—*Lives of Eminent American Physicians and Surgeons of the Nineteenth Century.* Edited by SAMUEL D. GROSS, M.D., Professor of Surgery in the Jefferson Medical College of Philadelphia. 8vo., pp. 836. Philadelphia: Lindsay & Blakiston, 1861.

BIOGRAPHY ranks among the most popular of studies. Almost every one is curious to know something of the domestic habits, the peculiar traits of character, and the events which marked the career of such as have distinguished themselves in the fields of science or of literature; in the arts which contribute to the safety, comfort, and happiness of man, or whose names have become identified with one or other of the departments of the world's history.

The life of the physician, in its even tenor, as ordinarily passed amid the seclusion of the study or within the privacy of the chamber of sickness and of suffering, furnishes but few incidents of a character to interest the general reader. This is true even of those members of the profession who have aided most largely in extending and enriching the science of medicine, and of rendering more exact and positive its ministrations, as well for the prevention as for the cure of disease.

The public, though benefited by the labors of the physician, in improvement of health and prolongation of life, to a much greater extent than they are aware, take small pleasure in hearing of his pursuits and daily cares; of the nature of the one and the sources of the other they can form but an imperfect conception. And yet the career of the physician is not, in every instance, without its heroism and its romance. There are to be found among the professors of the healing art a goodly number who, while risking their lives when deadly pestilence pervades the land in ministering to the wants of the down-stricken, in assuaging their sufferings, and affording them all possible protection from the destruction that smitest in darkness and wasteth at noonday, have exhibited more true courage, have confronted more actual danger, witnessed more thrilling and appalling scenes, and have performed a far greater and more positive amount of good to mankind than the conqueror in many a well-fought field.

The lives of nearly all the early physicians of our own country are replete with interest: many of them eminently so. They not only devoted themselves diligently and successfully to the legitimate practice of their profession amid, perhaps, the very pioneers of that advancing tide of immigration, destined to invade and people and bring into cultivation the remote plains and forests of the country, but became intimately associated with, if perchance they were not the originators of, whatever movements were made to augment the pecuniary, physical, moral, political, and religious well-being of the communities in whose midst they were located.

It was a fortunate circumstance, that so large a proportion of the earlier practitioners and teachers of medicine in this country were, in character, abilities, and mental culture, above the common standard. The major part of them possessed, in addition to fine natural abilities, all the advantages derivable from a thorough professional training at the most distinguished schools and hospitals of Europe. It was this which enabled them to secure for the American physician that high social position which he has ever since maintained. Removed, in a great measure, also, from the cramping influence which, in their day, the leading authorities in the several departments of medicine exercised over the entire profession on the other side of the Atlantic, and called upon to contend with disease amid communities placed under influences very dissimilar to those to which the inhabitants of Europe were subjected, and which, as might be expected, had the effect of modifying, in no inconsiderable degree, its character, phenomena, and course,—the early physicians of this country were compelled to rely, almost exclusively, upon their own observations and experience. Upon these they succeeded in basing a system of practice of the soundness and efficiency of which we have ample testimony. It is from the examples thus set them that the physicians who succeeded these pioneers of American medicine acquired much of that independence in theory and boldness of practice by which, to a very great extent, they are still characterized.

The biographies of very many of the early physicians of this country are yet to be written; while the materials necessary for their preparation are fast disappearing. They who will take upon themselves the labor of collecting such of these materials as may yet be procured, and of marshaling them in order, will receive the thanks of the profession, for preserving from oblivion the names of many of its members, who, by their characters and attainments, their industry and their skill, have conferred upon it equal honor and enrichment; while the public generally cannot fail to become interested in the narratives of lives that were spent exclusively in its service, and to acquire from them a more correct appreciation of the rightful claims the physician has upon its gratitude, protection, and support.

The names and labors of such men as Kearsley, Kuhn, Thomas Bond, Shippen, Redman, John Jones, Morgan, Ross, Currie, Dewees, Say, Parke, Tilton, Samuel P. Griffiths, Hutchinson, Annan, Seybert, and many more, deserve to be commemorated, as well for the credit of the profession of which they were distinguished members, as on account of what they so freely contributed toward the good of their fellow-creatures.

Few are the attempts that have been made to popularize the biography of American physicians and surgeons. Of the lives of many short notices may be found in the medical journals, and in the printed transactions of our State medical societies; while upon the death of one particularly distinguished for his position or his talents, there has been occasionally prepared, by special appointment, a memoir of greater fullness and pretension.

Almost the only systematic series of medical biographies, adapted to present a few of the more distinguished of the physicians and surgeons of our country in their true light to the notice of the general community, is that of Dr. James Thatcher, of Massachusetts. In this work, which was published in two octavo volumes, in the year 1828, a large mass of valuable facts, illustrative of the career, character, and professional labors of medical worthies, who flourished in the earlier period of the settlement of the United States and subsequently to the war of the Revolution, have been preserved from the oblivion to which they were fast descending, and arranged in a manner adapted to both interest and instruct.

Some years subsequently, Dr. Stephen W. Williams, also of Massachusetts, entered with great zeal upon the task of continuing the work commenced by Dr. Thatcher. As the result of his labors, he presented, in 1845, an octavo volume of some six hundred and fifty pages, comprising outlines, more or less extended, of the lives of American physicians who had died subsequently to the year 1828. From the energy, industry, and zeal evinced by Dr. Williams in the prosecution of his favorite theme, we have no doubt that, had he lived, he would have rendered more complete, by the additional materials he had accumulated, many of the biographical sketches already published, and supplied the lives of other physicians who, from their characters, their professional attainments, and successful labors, present strong claims upon our respect and gratitude.

The works just noticed, however valuable they may be esteemed, have by no means exhausted the subject, or occupied the entire field of American medical biography. The lives of many who were distinguished in their day for their varied and exact knowledge, and their practical skill as physicians and as surgeons, have not yet been told. Even of the physicians and surgeons whose names stand prominent upon the list of those who have most adorned, enlarged, and magnified the science and the art of healing in our midst, there are still wanting biographies through which the profession and the community may acquire a just estimate of the lives they lived and the works they performed—biographies in which, "without favor or prejudice," their social, moral, and intellectual worth is faithfully depicted; the eulogistic hyperboles of devoted friends and partisans, and the misrepresentations and detractions prompted by rivalry or envy being alike overlooked or corrected.

There is ample room for a new volume, or even volumes, devoted to the commemoration of the eminent departed physicians and surgeons of our land. All that is demanded to render such a work popular, in and out of the profession, is, that they who contribute the materials for its composition shall present, in a clear and simple narrative, the prominent events of the lives of those of whom they write, with an honest judgment of their characters as men, and of their standing in a professional point of view.

For the work before us we are indebted to the suggestion and promptings of Dr. Gross; it originated in a desire on his part to popularize the profession, and to place its services and claims more conspicuously, than had yet been done, before the American people. Not only does it comprise several contributions from his pen, but it has received throughout the benefit of his editorial supervision. It furnishes the lives of a number of those who have gained for themselves a high rank upon the roll of American physicians and surgeons, as well by their talents and skill as by the extent to which they have enriched and illustrated their profession and magnified it in public estimation. The lives are ably and candidly written, and present a very fair delineation of the career and standing of their respective subjects. There is, certainly, no very glaring attempt to extenuate their faults and shortcomings or to laud in excess their virtues and their worth.

The lives of Drs. Rush and Physick are among the most elaborate and discriminating of those embraced in the present series. The peculiar traits of character by which those gentlemen were respectively distinguished, their mental constitution and endowments, their professional merits, their labors in augmentation of the general fund of medical knowledge, and toward the perfection and extension of medical skill, are ably and faithfully delineated; and the particular relations which each of them held with respect to the community at large are indicated with truthfulness and candor.

No one can enter upon the perusal of either of the biographies referred to without becoming deeply interested; nor can any one rise from their perusal without having acquired a vivid idea of the two men of whom they tell,—their moral, intellectual, social, and professional standing.

Drs. Rush and Physick were, unquestionably, two of the most eminent of the earlier physicians of this country. By general consent, the rank of father of American medicine has been conferred upon the first, as that of father of American surgery has been upon the latter. To this honor they were, in all respects, justly entitled, from their talents, their industry and acquirements; the lustre they shed upon the American medical profession, the purity of their moral character, and the invariable amenity and dignity of their deportment, and the impulse which they gave, both by precept and example, to the careful cultivation and improvement in our midst of their respective branches. They taught their contemporaries and successors, as well physicians as surgeons, to rely less upon authority and precedent and more upon original observations and independence of thought in their investigation of the causes, nature, and treatment of diseases generally, and in their inquiry into the proper adaptation of chirurgical means to remedy the various accidents and injuries and deformities to which the human system is liable.

The eminence of Rush and Physick in their respective departments was not due to the comparatively small amount of professional knowledge and skill, requisite at the period when they flourished, to confer distinction, and the few among their contemporaries who were able or willing to contest with them for the mastery. No matter at what period they had commenced their professional career, earlier or later, they could not fail, with their peculiar qualifications, natural and acquired, to have attained the foremost rank among their professional contemporaries.

It is an error to suppose that Rush and Physick practiced and taught at a period when the medical profession of the country was composed mainly of ignorant or unskillful members, over whom pre-eminence was at best but an equivocal honor. On the contrary, from the commencement to the close of their career, there were numbered among their professional contemporaries many who were distinguished alike for their intellectual powers and their high professional attainments and skill; by several of whom their pre-eminence was very reluctantly and tardily acknowledged.

Dr. Rush was among the most voluminous of our older medical authors. Besides his "Medical Inquiries," in four large octavo volumes, we have his admirable work on the diseases of the mind; his notes to the works of Sydenham, Pringle, Cleghorn, and Hillary; and a volume of sixteen addresses, introductory to his courses of lectures in the University of Pennsylvania. In addition to his more strictly professional writings, Dr. Rush published a number of essays, literary, moral, and philosophical, most of which were collected and published in a volume in the year 1798.

Our earlier physicians published but little. The fact is, that previously to the termination of the first ten years of the present century, there was but little encouragement for the publication of medical works of any kind, beyond those employed as text-books by our more popular teachers in their public or private courses, or the few treatises which were considered, if not absolutely necessary, at least very useful, as books of reference in cases of doubt or emergency. For original medical works, by American authors, it may be said, without exaggeration, there was no demand. We rather think that there was entertained an opinion, both in and out of the profession, that the study of books was calculated to impair the skill of the physician,—to render him more of a theorist than a ready and skillful practitioner. We have been presented, nevertheless, with some able productions by our elders in the medical profession in this country. Nearly all of these appeared, however, either as detached papers or essays; and were either printed separately in pamphlet form, or appeared as contributions in one or other of the few professional journals then in existence.

Though presented in this humble way, these papers will be found replete with sound, practical sense. They evidence the close and careful habits of observation possessed by their authors, the cautiousness and general accuracy of their deductions, and the little bias they experienced, in their etiological, pathological, and therapeutic deductions, from the prevalent medical systems of their day. The style of these essays is, in general, uncommonly pure, clear, concise, and forcible. Were they collected and published in a form that would place them within the reach of the members of the profession generally, a very large proportion would be found, even at the present day, to be eminently instructive, more especially from the light they shed upon the causation, character, and course of the leading endemic maladies of America, and the history and nature of the several epidemics of disease that have here in the olden time, from period to time, prevailed. Such a publication would constitute a fitting monument to commemorate the talents, the industry, and public spiritedness of those who inaugurated in our midst the systematic study and practice of the healing art.

To become convinced of the distinguished character, the high professional attainments and elevated social standing of the compeers of Rush and Physick, it is only necessary to refer to those whose lives are recorded in the volume before us, and they constitute not a tithe of the eminent men who, contemporary with the "fathers of American medicine and surgery," adorned and magnified the medical profession in this country. Among the contemporaries of Rush and Physick, included in the list of those whom the volume of Dr. Gross commemorates, are such men as Samuel

Bard, James Thatcher, John Warren, Caspar Wistar, W. J. Macneven, Samuel L. Mitchill, Thomas C. James, Samuel Brown, David Hosack, and E. McDowell.

Dr. Samuel Bard, of New York, was three years older than Rush, and twenty-six years older than Physick; the former he survived eight years, while he preceded the latter to the grave by some sixteen years. Dr. Bard is better known to the physicians of the United States, whose professional studies were prosecuted during the early portion of the present century, by his very excellent compend of midwifery—a compend which, for the excellency of its arrangement and the clearness and accuracy of its teachings, has not been excelled, scarcely equaled, by any that has since appeared. The name of Dr. Bard is most frequently quoted, however, by medical writers, in connection with his treatise on the nature, cause, and cure of the "*angina suffocatoria, or sore-throat distemper*," which was originally published in the first volume of the Transactions of the American Philosophical Society. This treatise presents a most graphic description of the epidemic throat distemper which prevailed in certain parts of New York in 1771, showing most conclusively its entire identity with the epidemic diphtheria of the present time. It proves Dr. Bard to have been a close and accurate observer, and prepared to record readily his observations in language at once simple and chaste, but withal clear and forcible.

Dr. John Warren, of Boston, was eight years younger than Rush, but fifteen years older than Physick. He survived the first only two years, while the latter survived him some twenty-two years. Dr. Warren was equally distinguished for his classical attainments, his professional knowledge, his untiring industry, and his skill both as a physician and as a surgeon; for his high moral standing; his ardent patriotism, at a time when patriotism demanded the sacrifice of every personal interest for the common good; and for his unbounded social popularity.

Dr. Caspar Wistar, younger than Rush by sixteen years, and the senior of Dr. Physick by seven years, outlived the first five years, while the latter outlived him some nineteen years. As a teacher of anatomy, as a medical and surgical practitioner, and as a promoter and cultivator of general science, his name is widely and favorably known. With the exception of Drs. Rush and Physick, no public teacher commanded more fully the respect and esteem of his class than Dr. Wistar—none ever wound himself more effectually around the hearts of those who came in immediate contact with him. His deportment was always kind and conciliatory, and his manners invariably highly popular in the best sense of the term.

Dr. Samuel L. Mitchill, of New York, whose name ranks among the most active and prominent of the pioneers who devoted successfully their time and their talents to the promotion in America of medicine, in common with the natural sciences generally, was born in 1764, nineteen years before Rush, whom he survived eighteen years. He was four years older than Physick, and died six years before him. He was renowned for much and varied learning, for a genius, prompt in execution and original in combination, and for a rare simplicity of character. He was a most successful promoter of physical science. He was more, in fact, of a natural philosopher than a physician.

Dr. David Hosack, of New York, whom Dr. Currie designates as "the polite scholar, the learned and skillful physician, the sincere friend, and accomplished gentleman," was born twenty-four years after Rush, and one year after Physick. He survived the first twenty-two years, and preceded by two years the latter to the tomb. He published a number of essays on different medical subjects, replete with sound practical remarks. He is best known, however, by his monograph on the laws of contagion, in which is set forth and defended the doctrine that the contagion which produces yellow fever is a morbific principle generated beneath a tropical sun and imported into this country in the persons and clothing of the crews, in the air of the hold, and in the cargoes of infected vessels arriving from yellow-fever localities. This principle in a free, healthy, atmosphere he believed to be comparatively inert, but that it multiplied itself rapidly, and assumed an increased virulence in a hot, confined, and impure atmosphere.

Dr. Ephraim McDowell, one of the most distinguished, bold, and successful surgeons of the West during his day, and the first who ever performed the operation of ovariotomy, was sixteen years the junior of Dr. Rush, whom he survived twenty-seven years. He was three years only younger than Dr. Physick, and died seven years before him. He is represented as a fine classical scholar; while history and polite literature were the subjects which, outside the graver sciences more intimately connected with his own professional studies and pursuits, occupied most of his time and attention.

Dr. James Thatcher, one, perhaps, of the most voluminous among the earliest medical writers of our country, was seven years younger than Rush, and sixteen years older than Physick. He survived the first thirty-one, and the latter seven years. He was among those patriots who devoted themselves to the service of the country in the darkest period of her history. He was a man of science, and of extensive literary acquirements, and a diligent and successful practitioner of the healing art. Among his leading works may be enumerated a Journal of the Events of the Revolutionary War, issued in 1824, and a second edition in 1826; a new American Dispensatory, issued in 1810, and running through four editions in eleven years; a modern Practice of Physic, a first edition in 1817, and a second in 1821; a Monograph on Hydrophobia, in 1821; a volume of American Medical Biography, in 1828; an essay on Demonology, Ghosts, Apparitions, and Popular Superstitions, in 1831; a History of Plymouth, in 1832, and a second edition in 1835. In addition to these, he was the author of a number of valuable papers on medical, economical, and scientific subjects.

Dr. W. J. Macneven was born in 1763, and was, consequently, eighteen years younger than Rush, and five years the senior of Physick. He survived the first twenty-eight, and the latter four years. He was a man of liberal education, and a highly esteemed teacher of medicine in New York for upwards of twenty years, during which period he was constantly engaged in contributing, with zeal and ability, to the promotion of the best interests of his profession.

Dr. Thomas C. James, the polite scholar, the successful practitioner of obstetrics, and for many years the highly popular professor of midwifery and of the diseases of women and children, in the University of Pennsylvania, was born twenty-one years after Rush and two years before Physick. The former he survived twenty-two years, but died two years before the latter.

Dr. S. Brown, an esteemed medical practitioner and teacher in the West, one who sought by all honorable means to promote, assert, and sustain the dignity, vindicate the honor, and elevate the status of the medical profession, was born in 1769, twenty-four years after Dr. Rush, and one year after Dr. Physick. He survived the former seventeen years, while his death occurred one year previous to that of the latter.

Of the contemporaries of Rush and Physick whose memoirs are not included among those collected by Dr. Gross, there is a very long list, embracing names of individuals in no respect inferior in standing and in talents—no less earnest and untiring in their efforts for the enlargement of medical knowledge, and for the elevation in character, in educational preparation, and in skill, of the entire medical profession of our country, to those whose names are given above.

It would occupy too much space were we to attempt to notice severally the different biographies comprised in the volume under review. They have been prepared, all of them, with great ability, and evince much more candor and kindness, a far more just appreciation of character and professional worth, than, perhaps, we had a right to expect, when we take into consideration the large space in public attention most of them occupied in their lifetime; how nearly the period in which they flourished touches upon our own, too near to extinguish entirely the influence of the friendships and enmities, the partisanship and rivalry, they excited when they lived; and of the many circumstances, connected with the career of most of them, by which the formation of a just estimate of their professional standing and worth is rendered extremely difficult.

To those who experience the laudable desire to know something of the lives and services, the qualifications and the labors of the medical men to whose teachings, example, and suggestions is to be mainly attributed the present advanced condition, in this country, of medicine as a science and an art, we would recommend the work of Dr. Gross as one presenting superior claims to their notice, as well from the truthfulness of its narratives, as for the ability exhibited generally in its preparation. It cannot fail, we think, to be found holding a prominent position in every library in our country—most certainly in the library of every American physician.

